# lnvasive cystic hypersecretory carcinoma of the breast associated with papillary pattern: a rare and poorly recognised variant of ductal carcinoma of the breast

**DOI:** 10.3332/ecancer.2014.477

**Published:** 2014-11-04

**Authors:** Parul Gupta, Sonal Dhingra, Osman Musa, AN Srivastava

**Affiliations:** 1 Department of Pathology, Era’s Lucknow Medical College and Hospital, Lucknow 226003, India; 2 Department of Surgery, Era’s Lucknow Medical College and Hospital, Lucknow 226003, India

**Keywords:** breast, carcinoma, mastectomy

## Abstract

Cystic hypersecretory pattern is a rare and poorly recognised variant of invasive ductal carcinoma of the breast. Cystic hypersecretory lesions of the breast have a spectrum of morphological features ranging from clearly benign cystic hypersecretory hyperplasia (CHH), CHH with atypia, cystic hypersecretory carcinoma (CHC) to invasive CHC. Until now, no case of invasive CHC has been reported in India, to the best of our knowledge. We report a case of a 57-year-old female with a history of a lump in the inferomedial quadrant of the right breast for three years, gradually increasing in size. A mammography showed a well-defined, lobulated radio-opacity. A modified radical mastectomy was done. Gross examination showed multiple cystic spaces filled with thick gelatinous material and solid areas. On histopathology, cystic hypersecretory variant of invasive ductal breast carcinoma with focal papillary pattern was diagnosed. Cystic hypersecretory ductal carcinoma behaves in a low-grade fashion for many years but has a potential for invasiveness and metastasis, so regular follow-up of such cases is crucial.

## Introduction

Cystic hypersecretory carcinoma (CHC) and cystic hypersecretory hyperplasia (CHH) was first described in 1984 [1]. CHC is an uncommon distinctive variant of ductal breast carcinoma *in situ* that arises in the background of CHH and is characterised grossly by the presence of dilated ducts and cysts containing glistening, gelatinous material, and microscopically areas of micropapillary carcinoma in the epithelium lining the cyst [2]. CHC is thought to behave in an indolent manner but has the potential to give rise to invasive carcinoma [3]. An invasive component has been reported approximately in 20% of CHC cases, and it tends to be poorly differentiated ductal carcinoma with solid growth pattern and no secretory activity [2]. Positive reactions for periodic acid schiff (PAS), carcinoembryonic antigen (CEA), alpha-lactalbumin have been observed in the cyst contents, which are consistently negative for thyroglobulin. CHC are usually estrogen receptor (ER) and HER-2/neu positive [4]. There have only been a few cases of invasive CHC reported in the literature. We describe an additional new case of invasive CHC in a 57-year-old female, and the relevant literature is reviewed [Table 1].

## Case report

**Figure 1.**
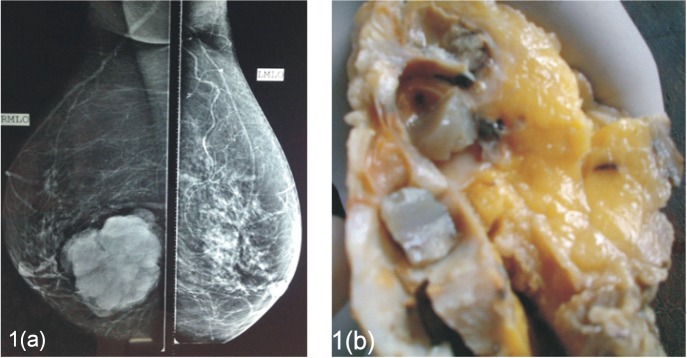
(a). Mammography, mediolateral oblique view showing well defined, lobulated radio-opacity in the right inferomedial quadrant. (b). The cut surface of the mass showing cysts filled with gelatinous secretions.

A 57-year-old female presented with a palpable mass in the inferomedial quadrant of the right breast. The lump was gradually progressive in size for the last three years. Physical examination revealed a painless, mobile, ill-defined, hard, 8 x 7 cm lump with no retraction of nipple, no nipple discharge, and no axillary lymphadenopathy. The patient had no history of benign breast disease previously or family history of breast cancer. A mammography showed a well-defined, lobulated radio-opacity in the inferomedial quadrant of right breast (Figure 1a).

**Figure 2.**
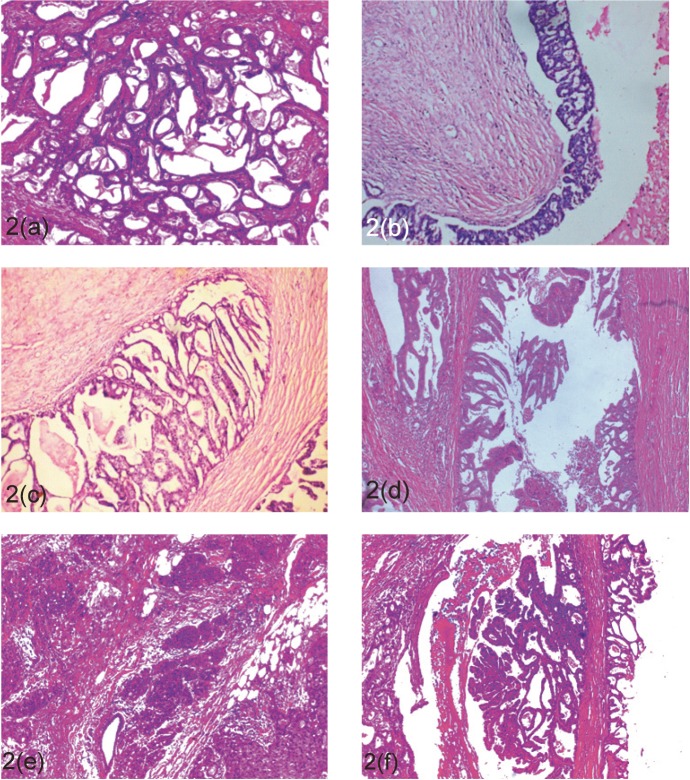
Microscopic findings (H & E). The lesion is composed of multiple cyst and ducts containing eosinophilic secretions (a; 40x). Some of the cysts are lined by flattened epithelium (a; 40x) while others show epithelial proliferations in form of pseudostratification, knobby tufts (b; 100x), Roman arch (c; 100x), to micro-papillary carcinoma *in situ* (d; 630X), and invasive pattern in form of solid sheets (e; 100x), and papillary pattern of ductal carcinoma (f; 100x).

The patient underwent a modified radical mastectomy. Grossly, the cut surface revealed a 7 x 7 cm tumour which showed multiple cystic spaces filled with thick gelatinous material and grey-white solid areas (Figue 1b). Microscopically, the tumour showed varied histological pattern with predominantly multiple variable- sized cystic spaces filled with PAS positive dense eosinophilic material resembling thyroid colloid (Figure 2a). These homogenous secretions were retracted from the surrounding epithelium producing a scalloped margin. The cyst lining epithelium exhibited a variable pattern that in most areas was flat to cuboidal (Figure 2a) and in other areas showed a proliferative change in the form of pseudostratification to knobby epithelial tufts (Figure 2b) to complex branching fronds that extend across duct lumen forming a Roman arch bridging pattern (Figure 2c). Areas of intraductal carcinoma (micropapillary type) (Figure 2d) accompanied by an invasive component comprising of solid pattern of moderately to poorly differentiated ductal carcinoma cells (Figure 2e) was seen. Some areas of tumour tissues also showed papillary pattern with fibrovascular core (Figure 2f). Immunohistochemistry (IHC) shows, the cystic contents were negative for CEA and thyroglobulin. Tumour was ER, PR, HER-2/neu negative.

A diagnosis of invasive cystic hypersecretory carcinoma was made with focal papillary pattern. None of the axillary lymph nodes were positive for tumour metastasis.

## Discussion

Rosen and Scott defined cystic hypersecretory carcinoma as a subtype of intraductal carcinoma of the breast and described this entity in a series of 10 cases [1]. Since then few authors have put this entity forward generally in the form of case reports and that further invasive CHC is much rarer (Table 1). Cystic hypersecretory lesions of the breast have a spectrum of morphological features ranging from clearly benign (CHH), a combination of benign and atypical epithelium (CHH with atypia), to cases that combine benign, atypical, and frankly malignant epithelium [5]. The characteristic findings of invasive CHC are formation of dilated ducts filled with eosinophilic colloid-like material in their lumens and lined by pseudostratified to micropapillary epithelium along with foci of invasion. Extravasation of cyst material into the stroma does not indicate invasion [1, 5, 11]. Invasion is heralded by solid nests of malignant cells and is usually poorly differentiated with no secretory characteristic. Among the reported cases of cystic hypersecretory breast lesion, most cases are of *in situ* CHC, with only a few cases of invasive CHC [Table 1]. So this makes our case extremely rare in the scenario that it is invasive CHC and also harbours foci of CHH and CHC *in situ* within the lesion and shows an additional morphology of papillary pattern. If no cytological atypia is present and the epithelium is flat or cuboidal, the lesion is characterised as CHH. Foci of the similar picture is seen in our case along with invasive areas and in accordance with long history of gradually progressing breast lump; it appears that the CHH has progressed to CHC *in situ* to invasive CHC showing the spectrum of evolution of this tumour within the same lesion.

The differential diagnosis of invasive CHC includes juvenile secretory carcinoma, mucinous carcinoma, malignant mucocele-like tumour, and metastatic thyroid carcinoma. Juvenile secretory carcinoma contains vacuolated cytoplasm and more bubbly secretions which are not typical features of CHC [22]. Mucinous carcinoma and malignant mucocele-like tumour also show cystically dilated ducts. However secretions of these lesions are rather pale and basophilic and do not show linear cracking artefacts, and the mucinous content are formed from extravasation of mucin within the stroma. Metastatic follicular thyroid cancer may mimic CHC, so a histological differentiation requires IHC stain for thyroglobulin [12, 13, 15]. CHC usually behaves in a non-aggressive manner but few reported invasive CHC cases, including our case, emphasise the need for follow-up and genetic studies for future risk assessment.

## Conclusion

CHC *in situ* of the breast is a rare distinctive variant of ductal carcinoma that behaves in a low-grade fashion for many years but, nevertheless, has a potential for invasive growth and development of distant metastasis. Under-diagnosis of CHC as a benign lesion that is CHH is a recognised phenomenon and should be avoided by extensive sampling of the lesion and looking for invasion. It is a persistent condition with the potential to evolve into carcinoma. Longer follow-up and study of additional cases will be necessary to determine if this lesion has distinctive clinical characteristics.

## Figures and Tables

**Table 1.** Review of cases of CHC in the literature.

Source, y	Age, y	Size/Location	Lymph Node Status	Type of Disease Present	ER/PR	Modality of Diagnosis and Final Therapy
Rosen and Scott [1], 1984	62	NA/L	N0	*In situ*	NA	MRM
	41	NA/R	N0	*In situ*	NA	MRM
	39	2.5 cm first/L	N0	*In situ*	NA	2 x Bx/MRM
	48	10 cm/R	NA	*In situ*	NA	SM
	78	8 cm/R	NA	*In situ*	NA	Bx
	‘old’	8 cm/R	NA	*In situ*	NA	SM
	52	‘large’/L	†	Invasive	Pos/NA	Bx
	47	2.2 cm/R	N1	Invasive	NA	MRM
	62	1 cm/L	N0	Invasive	NA	MRM
	34/55	NA/L	NA	*In situ*	NA	Bx
Guerry *et al* [5], 1988	NA	NA	N1	Invasive	NA	MRM
	NA	NA	†	Invasive	NA	MRM
17 additional cases			N0	*In situ*	NA	MRM
Colandrea *et al* [6], 1988	62/67	2 cm/R	N0Mx	*In situ*	Neg/neg	Cyto/2 x Bx/MRM
Adams and Lacey [7], 1990	70	4.5 cm/L	N0	Microinvasive	Neg/pos	Bx/MRM/RT/Tam
Kim *et al* [8], 1997	37	8.8 cm/R	N0	Invasive	NA	Cyto/MRM
Herrmann *et al* [9] 1999	49	6 cm/L	N0	Invasive	Pos/pos	Bx/MRM
Shah AK [10], 2000 (two cases)	NA	NA	NA	*In situ*	NA	NA
Park JM [11], 2002 (two cases)	NA	NA	NA	NA	NA	NA
Lee JS [12], 2004	45	4.7 cm/L	N0M0	Invasive	Neg/neg	Bx/MRM
Shin SJ [13], 2004						
Nine cases	42^**^	NA	N0	*In situ*	NA	Bx
One case	42^**^	NA	N (micro)	Invasive	NA	Bx (MRM)
Park C [14], 2004	49/L	NA	NA	*In situ*	NA	Bx
Resetkova E [15], 2005	NA	NA	NA	NA	NA	NA
Skalova A 2005 [16] (five cases)	66.8^**^	7–8 cm	NA	Three *in situ* Two invasive	One Case Pos/Pos	NA
Sahoo S [17], 2008	48/L	NA	NA	*In situ*	NA	Core needle Bx/SM
Chen DB [18], 2010	NA	NA	NA	Microinvasive	NA	NA
Song SW [19], 2011	43	NA	NA	Invasive	NA	Bx
D’Alfonso TM [20], 2014, Mean (ten case)	62.8^**^	0.9 cm^*^	NA	Nine *in situ*	7 pos/2 pos	NA
			NA	One microinvasive	Pos/pos	NA
Bi R [21], 2014 (three cases)	49.3^**^	NA	NA	One *in situ* Two invasive	1 pos/1 pos	MRM
Present case	57	7 cm	N0	invasive	Neg/neg	MRMs

* ER/PR indicates estrogen receptor/progesterone receptor; NA: not available; L: left breast; R: right breast; pos: positive; neg: negative; MRM: modified radical mastectomy; Bx: biopsy; SM: simple mastectomy; Cyto: cytology; RT: radiation; and Tam: tamoxifen; N (micro): lymph node micrometastasis.

** Mean age

† Indicates cases with distal metastatic disease.
